# Sodium Selenite Ameliorates Silver Nanoparticles Induced Vascular Endothelial Cytotoxic Injury by Antioxidative Properties and Suppressing Inflammation Through Activating the Nrf2 Signaling Pathway

**DOI:** 10.1007/s12011-023-04014-2

**Published:** 2023-12-27

**Authors:** Yunyun Ma, Lei Wang, Jing He, Xueping Ma, Jingjing Wang, Ru Yan, Wanrui Ma, Huiyan Ma, Yajuan Liu, Hongqian Sun, Xiaoxia Zhang, Shaobin Jia, Hao Wang

**Affiliations:** 1 General Hospital of Ningxia Medical University (the First Clinical Medical College of Ningxia Medical University), Yinchuan, 750004 Ningxia China; 2 Heart Centre &, Department of Cardiovascular Diseases, General Hospital of Ningxia Medical University, Yinchuan, Ningxia China; 3 School of Basic Medical Sciences, Ningxia Medical University, Yinchuan, Ningxia China; 4 Department of General Medicine, The First Dongguan Affiliated Hospital of Guangdong Medical University, Dongguan, China; 5 College of Traditional Chinese Medicine, Ningxia Medical University, Yinchuan, Ningxia China

**Keywords:** Silver nanoparticles, Sodium selenite, Vascular endothelium, Oxidative stress, Inflammation, Nrf2

## Abstract

Silver nanoparticles (AgNP) are the dominant nanomaterials in commercial products and the medical field, but the widespread occurrence of AgNP has become a global threat to human health. Growing studies indicate that AgNP exposure can induce vascular endothelial toxicity by excessive oxidative stress and inflammation, which is closely related to cardiovascular disease (CVD), but the potential intrinsic mechanism remains poorly elucidated. Thus, it has been crucial to control the toxicological effects of AgNP in order to improve their safety and increase the outcome of their applications.

Multiple researches have demonstrated that sodium selenite (Se) possesses the capability to counteract the toxicity of AgNP, but the functional role of Se in AgNP-induced CVD is largely unexplored. The aim of this study was to explore the potential protective effect of Se on AgNP-induced vascular endothelial lesion and elucidate the underlying mechanisms. An *in vivo* model of toxicity in animals was established by the instillation of 200 µL of AgNP into the trachea of rats both with (0.2 mg/kg/day) and without Se treated. *In vitro* experiments, human umbilical vein endothelial cells (HUVECs) were incubated with AgNP (0.3 µg/mL ) and Se for a duration of 24 h. Utilizing transmission electron microscopy, we observed that the internalization of AgNP-induced endothelial cells was desquamated from the internal elastic lamina, the endoplasmic reticulum was dilated, and the medullary vesicle formed. Se treatment reduced the levels of vascular cell adhesion molecule-1 (VCAM-1) and intercellular adhesion molecule-1 (ICAM-1), inhibited the release of pro-inflammatory cytokines (specifically tumor necrosis factor (TNF)-α, interleukin (IL)-1β and IL-6), improved endothelial cell permeability, integrity, and dysfunction, and prevented damage to the aortic endothelium caused by AgNP. Importantly, we found that Se showed the capacity against AgNP with biological functions in guiding the intracellular reactive oxygen species (ROS) scavenging and meanwhile exhibiting anti-inflammation effects. Se supplementation decreased the intracellular ROS release and suppressed NOD-like receptor protein 3 (NLRP3) and nuclear factor kappa-B (NF-κB) mediated inflammation within AgNP-intoxicated rats and HUVECs. The anti-oxidant stress and anti-inflammatory effects of Se were at least partly dependent on nuclear factor erythroid 2-related factor 2 (Nrf2). Overall, our results indicated that the protectiveness of Se against AgNP-induced vascular endothelial toxicity injury was at least attributed to the inhibition of oxidative ROS and pro-inflammatory NF-κB/NLRP3 inflammasome by activating the Nrf2 and antioxidant enzyme (HO-1) signal pathway.

**Supplementary Information:**

The online version contains supplementary material available at 10.1007/s12011-023-04014-2.

## Introduction

Silver nanoparticles (AgNP) are considered to be an effective broad-spectrum antimicrobial agent that is utilized in daily life products and biological systems [1, 2]. However, with the increased application of AgNP, many researches have reported that AgNP exposure can aggravate the progression of cardiovascular disease (CVD) dysfunction through multiple pathways [3, 4].

The vascular endothelial cells referred to a single layer of mononuclear flat epithelial cells that continuously cover the innermost surface of the vessel wall. Damage to endothelial cell structure and function is considered to be the initiating factor in the development of atherosclerosis [5]. The vascular endothelium is particularly vulnerable to the toxic effects of AgNP. For example, an *in vitro* study showed that short-term and low-dose AgNP exposure can be taken up by human umbilical vein endothelial cells (HUVECs) and adhere to the cell membrane and induce vascular endothelial (VE)-cadherin phosphorylation, which in turn disrupt vascular integrity and increase endothelial cells permeability [6]. Additionally, reactive oxygen species (ROS) generated under the exposure of AgNP could notably result in the damage of HUVECs [7]. However, few studies have systematically explored the toxicity of AgNP on vascular endothelial cells. Therefore, effective prevention and treatment of AgNP-induced endothelial dysfunction is urgently needed.

Selenium (Se) is part of an essential trace element in almost all biological systems with numerous biological activities, including strong antioxidant characteristics associated with the reduction of free radical generation, anti-inflammatory effects, and stabilization of the redox system [8]. Several studies have reported that Se was capable of protection against the toxicity of heavy metals, including its role against AgNP-induced toxication. For example, by inhibiting the development of oxidative damage and enhancing anti-inflammation, Se has significant potential to reduce AgNP-induced testicular toxicity and liver injury [9, 10]. Our previous study also showed that Se effectively prevented oxidative insults, alterations in mitochondrial dynamic imbalance, and ultrastructural reactions in the lung tissue and myocardium following AgNP exposure [11, 12]. Many investigations have demonstrated that Se activates the nuclear factor erythroid 2-related factor 2 (Nrf2) by contributing to decreased expression of inflammatory cytokines, oxidative stress, and regulating metabolism [13, 14]. However, with these benefits, the effect of Se on the dysfunction of the cardiovascular system under AgNP exposure whether mediated by activation of the Nrf2 signal pathway has not been described and requires further elucidation. Thus, our study aimed to explore the protective role of Se in AgNP-induced vascular endothelial inflammation and oxidative stress and its mechanisms through the Nrf2/HO-1 axis, which may be important for the design of new treatments targeting AgNP-induced endothelial impairment.

## Materials and Methods

### AgNP Material Characterization and Se Sample Preparation

AgNP of 20 nm sizes were obtained from US Research Nanomaterials, Inc. AgNP were dissolved and stored according to a previous method [15]. Before transmission electron microscopic examination (TEM) analysis, the stock suspension of AgNP was immersed in ultrapure water and underwent three rounds of ultrasonic oscillations at a low temperature for a duration of 30 min.

To obtain a 100 mM concentration of Se stock solutions, 0.1729 g of Se (Sigma, USA) was dissolved in 10 mL ultrapure water. Following this, the stock solution underwent dilution using the same medium, resulting in a 1 mM working solution, which was subsequently stored at −20 °C.

### Experimental Animals and Intervention

The Medical Laboratory Animal Center of Ningxia Medical University provided forty identical, male Sprague-Dawley (SD) rats weighing between 220 and 240 g. In line with the established feeding standard, each rat was confined to standardized cages under a 12-h light/dark cycle and exposed to standard temperature and relative humidity levels ranging from 54 to 59%. These animals were provided with a standard commercial maintenance diet (purchased from Jiangsu Xietong, China; ingredients: 11.1% fat, 67.4% carbohydrate, 21.5% protein) and unrestricted access to water.

The dietary nutrients in this study were formulated according to GB14924.3 (Table S1). In our study, all animal experiments were conducted in accordance with institutional and national guidelines for the care and use of laboratory animals. The study received approval from the Laboratory Animal Ethical and Welfare Committee of Ningxia Medical University (IACUC-NYLAC-2022-202).

In order to examine the protective effect of Se on AgNP-induced damage to the endothelial cells of the aorta, the animals were randomly divided into 4 groups following a 1-week acclimatization period: (1) Control group (Control): rats were administered 200 µL of saline solution via intratracheal instillation; (2) AgNP intervention group (AgNP): each rat was treated with 200 µL (1 mg/mL) AgNP by tracheal perfusion with only once; (3) Selenium group (Se): sodium selenite dissolved in distilled water (0.2 mg/kg/day) was intraperitoneally administered for 7 days; (4) AgNP+Selenium group (AgNP+Se): each rat received 200 µL AgNP by tracheal perfusion for only once and simultaneously treated with an equal volume of Se. The doses of Se and AgNP were determined based on previous studies that had successfully established the experimental model [12, 16]. Body weight was recorded every 3 days. After 2 weeks, all of the rats were anesthetized with isoflurane and subsequently euthanized. Blood samples were collected through cardiac puncture for serum preparation, while the aorta was isolated, cleaned using a 0.9% NaCl solution, ultimately stored for subsequent histopathological and biochemical analyses, including the assessment of biomarkers related to oxidative stress and inflammation gene expression.

### HUVECs Culture

The American Laboratory of Sciences (Catalog 8000) provided the HUVECs, which were then cultured in a specific medium for endothelial cells (ScienCell #1001). The medium was added with 5% heat-inactivated fetal bovine serum (FBS), 1% penicillin/streptomycin, and 1% endothelial cell growth factors (ECGS). Once the culture reached a 70–80% confluence, the cells were utilized for subsequent investigations.

### Cell Viability Assay

The evaluation of cell viability was carried out by utilizing the Cell Counting Kit-8 (CCK-8) (AbMole, China) according to the procedures outlined by the manufacturer. HUVECs were cultivated in 96-well plates until they reached the desired confluence. Subsequently, they subjected to various treatments and were incubated at 37 °C for a duration of 24 h. Then each well was added with 10 µL of CCK-8 and allowed to incubate for an additional 2 h. The absorbance was then determined employing 450 nm (ALLSHENG, Hangzhou, China).

### Transmission Electron Microscopy (TEM) Examination

The aortic tissues were fixed in a solution containing 2% glutaraldehyde for a period of 2 h. After this, they underwent three consecutive washes with 0.1M dimethyl sodium arsenate at intervals of 2 h. Afterwards, the tissues were post-fixed for 2 h in a solution containing 4% osmic acid and then rinsed twice with 0.1M dimethyl sodium arsenate. To aid in the dehydration process, a series of escalating alcohol concentrations was applied to the tissues. It should be noted that all the aforementioned procedures were conducted at a temperature of 4 °C. Following the completion of the dehydration process, the samples were permeated with propylene oxide and then embedded in an epoxy resin. The subsequent step involved polymerizing the resin at 60 °C for 48 h. To achieve ultra-thin sections, a diamond knife was used for sectioning purposes. These sections were subsequently stained using uranyl acetate and lead citrate. After the preparation process, we observed these sections and captured corresponding images using a TEM.

### Histopathological Examination

First, the samples were fixed in 4% paraformaldehyde (PFA) at 4 ℃ for 24 h, then dehydrated in different concentrations of ethanol solutions (100%, 95%, 90%, 80%, and 70%) and embedded in paraffin wax after xylene transparency. Second, sections were then cut at 4 µm using a rotary slicer. Sections were examined microscopically by hematoxylin and eosin (H&E) staining and Masson’s trichrome staining.

### Determination of Oxidation Indexes

After the treatment, blood specimens extracted from diverse groups were used to centrifugate at 3000 g for a duration of 10 min for the isolation of plasma. The levels of superoxide dismutase (SOD), glutathione (GSH), and malondialdehyde (MDA) in plasma were quantified using specialized biochemical reagent kits obtained from a commercial company (Nanjing Jiancheng, China).

### ROS Measurement *In Vivo* and *In Vitro*

ROS levels were detected by DCFH-DA according to test kits (Beyotime, China). For the *in vitro* experiments, HUVECs were initially cultured in 24-well plates with 3 × 10^4^ cells/well. The cells with a confluence of 70–80% were treated with Se and AgNP for 24 h. Subsequently, the cells were washed and incubated for 30 min at 37 °C in the presence of 10 µM DCFH-DA, ensuring darkness during the incubation. For the *in vivo* investigations, it is essential to first dewax the tissue samples. Subsequently, the dewaxed tissue should be rinsed 3 consecutive times with phosphate-buffered saline (PBS), each rinse lasting for a duration of 5 min. To proceed further, 100 µL of a 10 µM solution of DCFH-DA should be supplemented onto each aorta tissue. Following this, the solution should be incubated at a temperature of 37 °C in darkness for an additional 30 min. After the incubation period, the tissue slices need to be rinsed thrice with 1 × PBS for 5 min each time. To complete the staining process, a fluorescent sealing agent containing 4′,6-diamidino-2′-phenylindole (DAPI) should be added, and a cover glass should be used to seal the samples. Finally, the sections should be observed using an Olympus fluorescence microscope, and the resulting images should be collected for further analysis.

### Immunohistochemistry and Immunofluorescence Staining

The tissues were prepared with an approximate thickness of 4 µm, and the paraffin surrounding the aortic tissues was eliminated using xylene. Then, the tissues underwent hydration using a range of ethanol concentrations (100%, 95%, 80%, and 70%) with each concentration lasting 5 min. Tissues were washed with PBS for 3 times at room temperature. Subsequently, the tissues were permeated with 0.5% Triton X-100 for 30 min, citrate antigen retrieval solution for 15 min, and finally blocked with goat-derived rabbit serum (Nakasugi Jinqiao, China) for 40 min. After the blocking step, the tissues were incubated overnight at 4 °C with primary antibodies ICAM-1, VCAM-1, and NLRP3 separately for immunohistochemistry. Later, the tissues were treated with biotinylated secondary antibody at room temperature for 1 h. After washing with PBS 3 times, the tissue slices were exposed to diaminobenzidine (DAB) stock solution for 3 min and counterstained using hematoxylin for 2 min, resulting in blue-stained nuclei. For immunofluorescence, the sections were incubated overnight at 4 °C with anti-ZO-1 (1:200; Affinity), anti-VCAM-1 (1:100; Abmart), anti-NLRP3 (1:100, Abmart), anti-Nrf2 (1:100, Affinity), and anti-CD31 (1:1000, Servicebio), respectively. After rinsing with PBS 3 times, the sections were separately incubated with goat anti-rabbit lgG /TRITC (1:200, ZSGB, China) or goat anti-mouse lgG /FITC (1:200, ZSGB, China). Following another round of washing with PBS 3 times, the sections were sealed with DAPI and captured using a fluorescence microscope (OLYMPUS, Japan). The software Image J was utilized to determine the percentage of the immune-stained areas.

### Preparation of Thoracic Aortic Rings

The isolated thoracic aorta was placed in a clean petri dish and injected with saline to remove the residual blood in the aorta. Then, the fat and connective tissue surrounding the aorta were then separated under a dissecting microscope. The isolated aorta was transected with straight scissors into a 4-mm long loop, and placed in 96-well plates with ABW^®^ matrigel, as well as treated with AgNP and Se in a 37 °C incubator. The fluid was changed every 2 days. The number of budding rat aortic cyclic neovascularization was observed and counted on day 4 under a high-power inverted microscope.

### The *In Vivo* Measurement of Vascular Permeability

Evans blue is a highly water-soluble synthetic diazo dye with a strong affinity for serum albumin and is a high molecular weight protein tracer in the blood. Under normal conditions, lower plasma albumin cannot penetrate the blood-brain barrier and is therefore often used to measure the integrity of the blood-brain barrier. We assessed vascular permeability in rat heart by slow injection of 1% Evans blue solution (2 mg/kg) through the tail vein of rats [17]. Changes in the skin and mucosal membranes of the rats were observed, and representative photographs were taken. After 2 h, all the rats were sacrificed simultaneously as soon as possible. Subsequently, saline was then injected to eliminate any remaining traces of blood along with the Evans blue solution within the cardiac region. The excised hearts were collected and placed in a 2.0 mL centrifuge tube with 1 mL of normal saline per 100 mg of tissue. The tissues were rapidly ground using a tissue homogenizer to make a homogenate and then subjected to 1000 g centrifugation for 20 min in a 4 °C ultracentrifuge. Following that, two portions of formamide were introduced to each tissue homogenate and subsequently immersed in a water bath maintained at 37 °C for a duration of 24 h. This incubation period aimed to extract Evans blue from the tissues. The quantity of Evans blue present in the heart was determined using a UV-spectrophotometer, specifically measuring the absorbance at 620 nm.

### Endothelial Tube Formation

An 80 µL volume of growth factor-reduced matrigel (ABW, China) was used to coat a 96-well cell culture plate, which was subsequently incubated at 37 °C for 30 min to facilitate matrigel polymerization. Following this, isolated primary HUVECs were seeded onto the wells coated with matrigel, and they were then cultured at 37 °C for 6 h in the presence of either AgNP (0.3 µg/mL) alone or both AgNP (0.3 µg/mL) and Se (6 µM) to observe tube formation. Tube formation was documented using an inverted microscope and analyzed utilizing the Image J software.

### Endothelial Cell Migration

To evaluate the impact of AgNP on the movement and penetration of endothelial cells, transwell permeable supports from Corning Incorporated (NY, USA) were employed. Initially, a serum-free medium containing 3 × 10^4^ HUVECs was introduced into the upper chamber of the transwell system using 200 µL. Following that, the HUVECs were exposed to 0.3 µg/mL AgNP and either 0 µM or 6 µM Se for stimulation purposes. After a 24-h incubation period, the cells were fixed with 4% paraformaldehyde for 30 min and subsequently stained with 0.1% crystal violet for 10 min. The chambers were placed there after rinsed with PBS, while any nonmigratory or invasive cells were gently wiped off from the upper side of the chamber using a cotton bud. The migratory cells were digitally captured under an inverted microscope (OLYMPUS, Japan) at five randomly selected fields. To count the cells, the Image J software was utilized.

### Quantitative Real-Time Polymerase Chain Reaction (qRT-PCR) Analysis

Real-time quantitative PCR was utilized to analyze the mRNA levels of ICAM-1, VCAM-1, endothelial nitric oxide synthase (eNOs), IL-6, TNF-α, IL-10, IL-1β, Nrf2, HO-1, NLRP3, ASC, caspase-1, and IL-18 in accordance with the provided guidelines, and the RNA extraction kit was employed to isolate total RNA from the rat aorta tissue (Corning, USA). A transcript uni cDNA was synthesized by a commercial PCR kit (TransGen, China). The 2^−ΔΔCt^ method was employed to cascade the relative mRNA levels, with GAPDH serving as the internal reference. Table 1 exhibits the primer sequences implemented in the present study.

**Table 1:** Target and reference gene primers used for Q-PCR

Target	Forward primers (5′-3′)	Reverse primers (5′-3′)
ICAM-1	TGTCAAACGGGAGATGAATGG	CTGGCGGTAATAGGTGTAAATGG
VCAM-1	TGAACCCAAACAAAGGCAGAGTA	TTGGGAGTTGGAAAACCATCAC
eNOs	GGTATTTGATGCTCGGGACTGC	GTGATGGCTGAACGAAGATTGC
Nrf2	AATTGCCACCGCCAGGACT	TCAAACACTTCTCGACTTACCCC
HO-1	CAGCATGTCCCAGGATTTGTC	CCTGACCCTTCTGAAAGTTCCTC
NLRP3	GATTTCTCCACAACTCACCCAA	AGTCTGGAAGAACAGGCAACAT
ASC	CTGTGCTTAGAGACATGGGCA	AGGGACACTGGTTGCAGTAG
Caspase-1	TGCCTGGTCTTGTGACTTGGAG	TGTCCTGGGAAGAGGTAGAAACG
IL-18	AACAGCCAACGAATCCCAGAC	TTGTTTTTACAGGAGAGGGTAGACA
TNF-α	CCAGGTTCTCTTCAAGGGACAA	GGTATGAAATGGCAAATCGGCT
IL-6	AGGATACCACCCACAACAGACC	TTGCCATTGCACAACTCTTTTC
IL-1β	TGACCTGTTCTTTGAGGCTGAC	CATCATCCCACGAGTCACAGAG
IL-10	AGAAGCTGAAGACCCTCTGGATA	TTCATTTTGAGTGTCACGTAGGC
GADPH	CTGGAGAAACCTGCCAAGTATG	GGTGGAAGAATGGGAGTTGCT

### Enzyme-Linked Immunosorbent Assay (ELISA)

In this study, we utilized rat enzyme-linked immunosorbent assay (ELISA) kits from Proteintech (USA) to determine the levels of IL-6, TNF-α, IL-1β, and IL-10 as inflammatory mediators in the serum samples. Moreover, a commercial human ELISA kit from Abmart (China) was used to measure the same inflammatory factors in the cell culture supernatants.

### Western Blot

Proteins were extracted from the thoracic aorta through homogenization using cold RIPA lysis buffer, and total proteins were quantified using a BCA kit (KeyGen, China). An equal amount of total protein was then subjected to 10% sodium dodecyl sulphate polyacrylamide gel electrophoresis (SDS-PAGE) and the resulting proteins were transferred onto PVDF membranes via electro transfer at a constant current of 200 mA for 45 min in the tris glycine methanol buffer. Then, the membranes were obstructed using 5% non-fat milk for a duration of 2 h at room temperature and subsequently incubated at a temperature of 4 °C in the presence of primary antibodies, rabbit or mouse lgG raised against NLRP3 (1:1000, Abmart), Caspase-1 (1:000, Abmart), ASC (1:1000, Abmart), TLR4 (1:1000, Abmart), NF-κB (1:1000, Abmart), HMGB1 (1:1000, Abmart), TNF-α (1:1000, Servicebio), IL-1β (1:1000, Servicebio), IL-18 (1:1000, Abmart), and β-actin (1:5000, Servicebio) overnight. After that, the membrane underwent 3 successive rinses for a duration of 10 min each with buffered saline containing tween (TBST). Subsequently, the membrane was incubated for 1 h with secondary HRP-labeled goat anti-mouse/rabbit lgG (1:5000, ZSGB, China). Ultimately, after washing, the membrane was exposed to ECL substrate for detection (NCM, China). Utilizing ImageJ software, the western blots were subjected to analyses concerning band size and density. Throughout all experiments, β-actin served as the internal reference.

### Statistical Analysis

Statistical analysis was performed using Prism 8.01 software (GraphPad Software Inc., USA). To assess the statistical difference between experimental groups, a two-way analysis of variance (ANOVA) was conducted, followed by the Turkey multiple-comparison test. *P* < 0.05 was statistically significant.

## Results

### Se Treatment Attenuated AgNP-Induced Morphological Damage in Rat Lung and Aortic Tissue and HUVECs

**Fig. 1:**
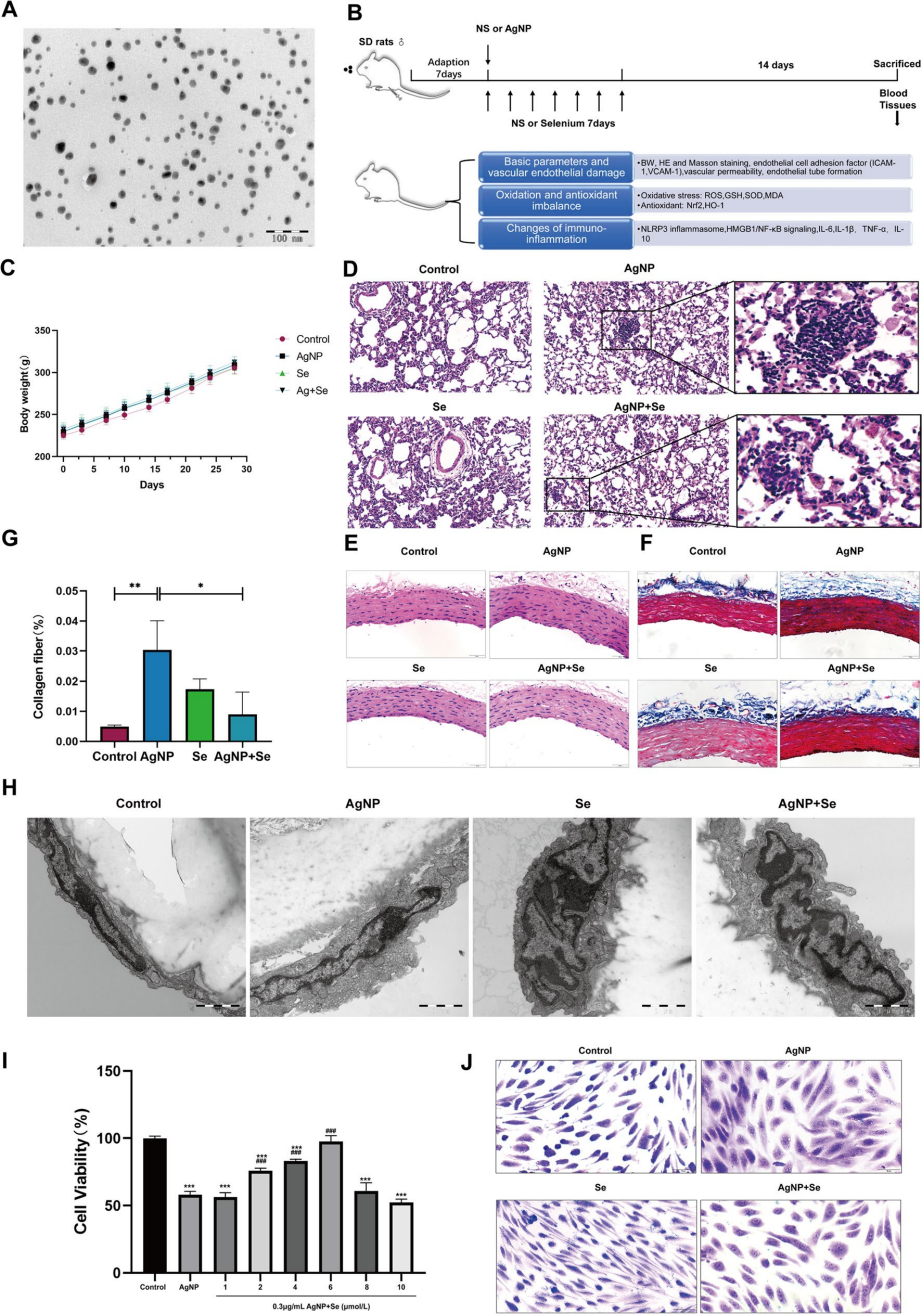
Se supplementation attenuated morphological damage to the endothelium of the lung and aorta induced by AgNP in rats and HUVECs. **A** Characterization of powdery silver nanoparticles with transmission electron microscopic (TEM). **B** Schematic diagram of the study. **C** Body weight. **D** H&E staining of lung tissue (bars =100 µm). **E** Histopathological alterations in the aorta were observed through light microscopy after performing H&E staining (bars =50 µm). **F** Masson’s staining was used to evaluate the accumulation of collagen in tunica media of aortas, the collagen fibers stained in blue (bars =50 µm). **G** Evaluation of collagen fibers area on the thoracic aorta. **H** Ultrastructural changes in the endothelium observed by 30,000×TEM. **I** Cell viability of HUVECs was cultured with 0.3 µg/mL AgNP at different concentrations of Se (0, 1, 2, 4, 6, 8, and 10 µm) for 24 h and was separately tested by CCK-8 assay (*n* = 3 per group). **J** Morphological changes of HUVECs by crystalline violet staining (bars =200 µm)

At the initiation of the experiment, TEM assay was employed to verify that AgNP in the water-based solution were spherical, well dispersed, and approximately 17.63 ± 3.35 nm in diameter (Fig. 1A), suggesting that this characteristic nanoparticles of Ag was capable of modeling in subsequent cytotoxic *in vivo* and *in vitro* research. *In vivo* rat experiment, the effects of Se intervention on this AgNP exposure-induced damage were evaluated **(**Fig. 1B). After a 2-week exposure of AgNP, the body weight showed a gradual increase, but with no significant difference among diverse groups, demonstrating that AgNP consumption or Se supplementation showed no apparent impact on body weight (Fig. 1C). Representative micrographs of histopathological changes in the lung measured by pathological H&E staining are shown in Fig. 1D. In the control and Se groups, a normal lung tissue structure was observed with no inflammatory cell infiltration. However, the alveolar septa thickening, alveolar lumen reduction, and a significant increase in alveolar macrophages were found in the AgNP group (Fig. S1). Importantly, the inflammatory cell aggregation was markedly decreased after Se supplementation, indicating that successful modeling injury described by previous reports could be ameliorated by Se intervention. Moreover, the thoracic aorta measured by H&E and Masson’s staining presented no apparent pathological changes between the control group and Se group (Fig. 1E–G). However, it could be clearly observed that the smooth muscle cells were disordered and the degree of fibrosis was higher in the AgNP group than in the control group, while Se treatment clearly alleviated the histopathological injury induced by AgNP (*P* < 0.05). Figure 1H shows the ultrastructure of aortic tissues in each group. In the thoracic aorta of control and Se groups, the endothelial cells were tightly adherent to the endothelium, whereas the endothelial cells in the aorta with the exposure of AgNP were desquamated from the internal elastic lamina, the endoplasmic reticulum was dilated, and medullary vesicle formation was evident. However, Se treatment attenuated the aforementioned damage to the cell structure induced by AgNP. To further identify the effects of Se on AgNP-induced endothelial cell injury *in vitro*, HUVEC cells were cultured with a range of different concentrations of AgNP (0, 0.05, 0.1, 0.2, and 0.3 µg/mL). The cytotoxicity of AgNP against HUVECs showed a clear dose-response relationship. Compared to the untreated group, cell viability significantly decreased to 54.6 ± 4.5% in 0.3 µg/mL of AgNP (*P* < 0.001, Fig. S2). Therefore, the concentration of 0.3 µg/mL AgNP was chosen as the optimal concentration for the subsequent study. Furthermore, we found that the treatment with Se at 1, 2, 4, 6, 8, and 10 µM for 24 h was not able to significantly inhibit the viability of HUVECs (Fig. S3), which aligned with a previous study [18]. When Se was combined with AgNP as shown in Fig. 1I, the treatment with 2, 4, and 6 µM of Se for 24 h reversed AgNP-induced inhibition of cell viability in HUVECs in a dose-dependent manner. The presence of 6 µM Se resulted in a level of cell viability that was nearly equivalent to the normal control group (100.0 ± 1.563). On this basis, we investigated the morphological changes of AgNP on HUVECs for the first time. Crystal violet staining results indicated that the HUVECs cells in the AgNP group were slightly stained and numerous vacuoles appeared in the cytoplasm, which could be restored by Se administration (Fig. 1J). Collectively, the above findings confirmed the ability of Se to protect vascular endothelial cells against AgNP damage.

### Se Treatment Reduced Vascular Endothelial ICAM-1 and VCAM-1 Expressions Increased the Level of eNOs

**Fig. 2:**
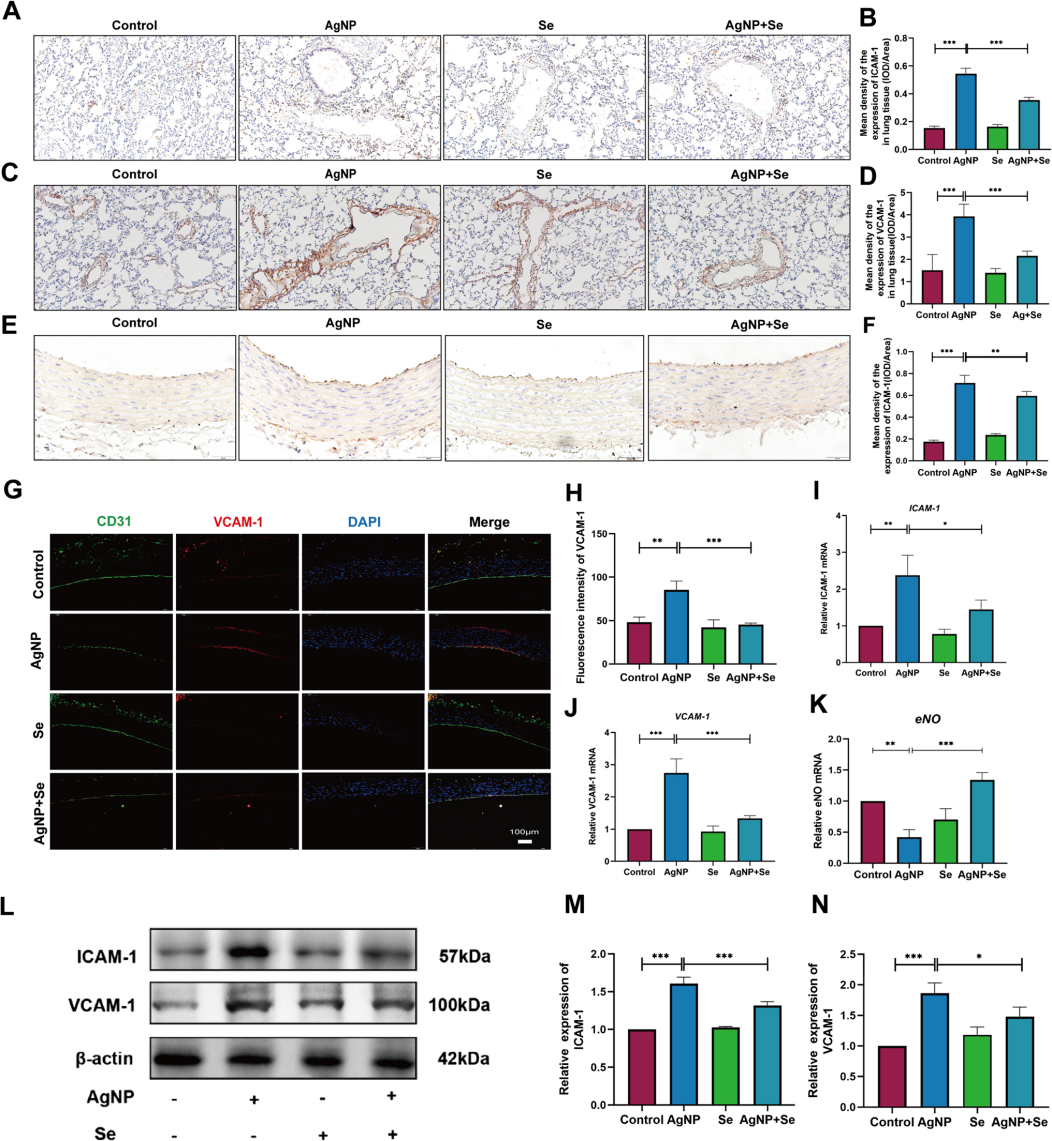
Se treatment attenuated AgNP-induced expressions of the pulmonary and aortic vascular endothelial adhesion factors ICAM-1, VCAM-1. **A**–**D** Representative photomicrographs of immunohistochemistry and quantitative analysis showing the detection of ICAM-1 and VCAM-1 in the lung tissue, the positive areas were stained in brown (bars =100 µm). **E**, **F** Representative photomicrographs of immunohistochemistry and quantitative analysis showing the detection of ICAM-1 in the aortic tissue, the positive areas were visualized through brown staining (bars = 50 µm). **G** Dual immunofluorescence staining for the co-expression of VCAM-1 and CD31 in the aortic endothelial cells as visualized in the images. Blue, nuclei; red, VCAM-1; green, CD31 (bars = 50 µm). **H** Fluorescence intensity analysis of the VCAM-1 in aortic tissue. **I**–**K** The mRNA expression levels of ICAM-1, VCAM-1, and eNOs in aortic samples were analyzed by qRT-PCR. **L** Representative western blot images and the protein levels of ICAM-1 and VCAM-1 in aortic tissue (**M**, **N**). Values are mean ± SEM and obtained from 3 independent experiments. ^*^P < 0.05, ^**^P < 0.01, ^***^P < 0.001

Overexpressions of adhesion molecules ICAM-1 and VCAM-1, representative indicators of abnormal vascular endothelial function, were separately determined by immunohistochemistry and immunofluorescence, and found with remarkable increases in the lung (Fig. 2A–D) and thoracic aorta (Fig. 2E–H) of rats exposed with AgNP, compared with those in the control group. In contrast, Se intervention significantly decreased the levels of ICAM-1 and VCAM-1 compared to the AgNP group. Consistently, similar changes of ICAM-1 and VCAM-1 were identified by qRT-PCR (Fig. 2I, J). Additionally, Se treatment notably increased the mRNA expression of eNOs in AgNP-induced aortic tissues (*P* < 0.001) (Fig. 2K). These mRNA levels of results were in parallel with further validation of protein expressions by western blot (Fig. 2L–N). Taken together, these findings indicated that Se had the potential therapeutic effect to improve the damage of vascular endothelial cells by AgNP.

### Se Treatment Improved Vascular Permeability and Dysfunction in AgNP-Administered Rats and HUVECs

**Fig. 3:**
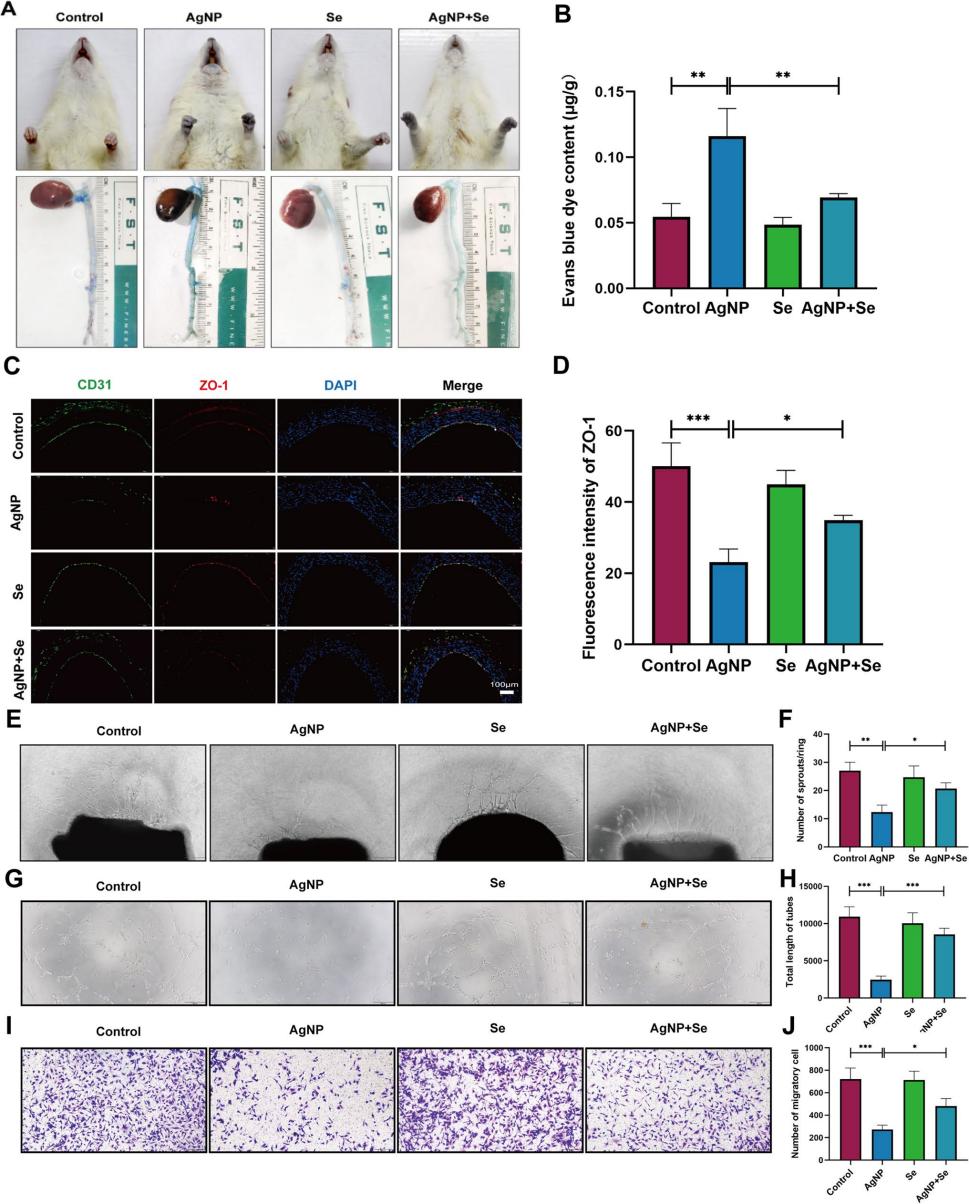
Se treatment improved AgNP-induced vascular endothelial permeability and dysfunction *in vivo* and *vitro*. **A** Evans blue extravasation in heart tissue and representative images of rat heart and mucosa. **B** Quantitation of Evans blue extravasation in heart tissue. **C** Dual immunofluorescence staining for co-expression of ZO-1 and CD31 in the aortic endothelial cells. Blue, nuclei; red, ZO-1; green, CD31 (bars = 100 µm). **D** Fluorescence intensity analysis of the ZO-1 in aortic tissue. **E** Inverted phase contrast microscopy of arteriolar rings that have budded as well as grown branches within the matrigel after grouping interventions (bars = 200 µm). **F** Statistical analysis of arterial ring sprouting and total length of branch. **G**, **H** To evaluate the angiogenesis ability of HUVECs, the tube formation assay was performed in the presence of 0.3 µg/mL AgNP with or without Se for 24 h. The tube length was quantified and shown on the right (bars = 50 µm). **I**, **J** The extent of migration was measured by determining the area covered by crystal violet-positive cells (*n* = 3 per group, bars = 200 µm). Values are mean ± SEM and obtained from 3 independent experiments. ^*^P < 005, ^**^P < 0.01, ^***^P < 0.001.

In order to determine whether Se intervention has protective effects on vascular permeability leakage in rats treated with AgNP, we conducted an experiment involving the injection of Evans blue dye and observed its leakage from plasma into the interstitial space. The skin around the nose, mouth, and paw tissues of AgNP-treated rats exhibited a noticeable blue coloration in comparison to the control group (Fig. 3A), and the levels of Evans blue were considerably higher in the hearts (Fig. 3B) and lungs (Fig. S4), clearly indicating that AgNP exposure caused an increase in vascular permeability (*P* < 0.01). However, it was observed that the administration of Se significantly reduced vascular permeability when compared to the rats in the AgNP group (*P* < 0.01). In addition, the downregulation of intercellular tight junction zonula occludens (ZO)-1 usually represents increased endothelial permeability in vascular dysfunction. To further assess the effect of AgNP on endothelial dysfunction, as shown in Fig. 3C and D, through the use of ZO-1 and CD31 immunofluorescence co-staining, our research revealed that the AgNP-exposure exhibited a remarkable decrease in ZO-1 expression compared to the control group (*P* < 0.001). However, the fluorescence intensity of ZO-1 in the thoracic aortic endothelium of Se-treated rats was significantly higher when compared to the AgNP group (*P* < 0.05). These findings strongly indicated that AgNP potentially induced a decline in ZO-1 levels within aortic endothelial cells, consequently leading to an elevation in vascular endothelial permeability. We further evaluated the effect of AgNP on angiogenesis by rat aortic ring experiment. As shown in Fig. 3E, compared to the control group, only a few shorter buds were formed in the aortic ring after exposure to AgNP, and the total length of the aortic ring bud was significantly reduced (*P* < 0.01) (Fig. 3F). The inhibitory effect of AgNP on the formation of new micro-vessels was notably diminished following treatment with Se, and there was a significant increase observed in the total length of aortic ring budding as compared to the AgNP-exposed group (*P* < 0.05). Similar results were also found in HUVECs, and AgNP exposure resulted in a notable decline in angiogenic ability (Fig. 3G, H) and cell migration (Fig. 3I, J) as compared to the control group. In contrast, the administration of Se effectively attenuated the detrimental effects induced by AgNP exposure. These results demonstrated that Se intervention was beneficial in counteracting AgNP-induced cytotoxicity and mitigating cell dysfunction. All the above data and images indicated that AgNP exposure could affect vascular integrity, increase permeability, reduce vascular regeneration, and impair endothelial barrier function, suggesting that Se administration had a protective effect on AgNP-induced vascular dysfunction.

### Se Treatment Negatively Regulated the Inflammatory Factors Induced by AgNP Exposure in Rats and HUVECs

**Fig. 4:**
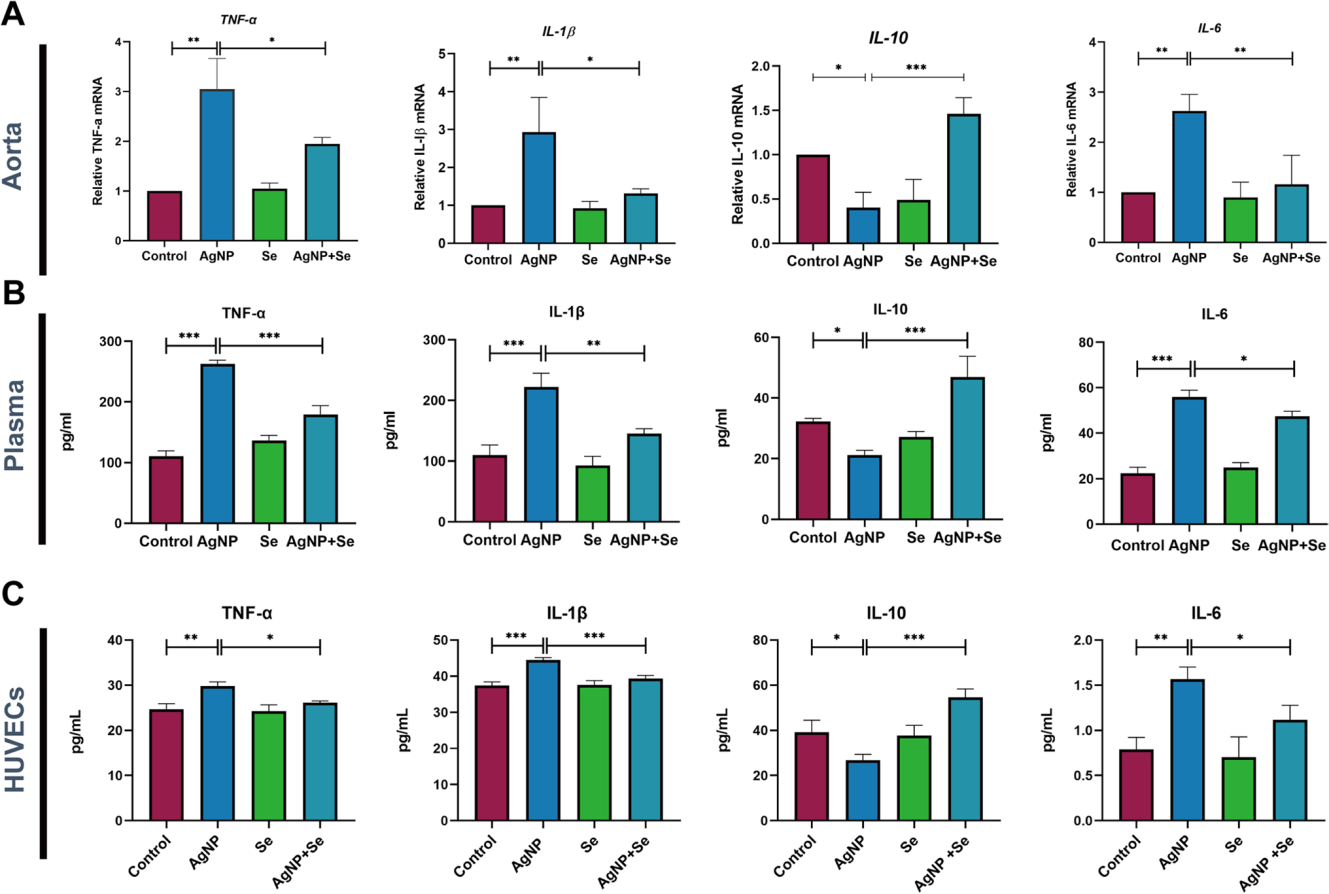
Se treatment suppressed the levels of inflammatory factors induced by AgNP exposure in rats and HUVECs. **A** qRT-PCR was performed to analyze the relative mRNA levels of IL-1β, TNF-α, IL-6, and IL-10 in aortic tissues. **B** ELISA was employed to quantify the release of TNF-α, IL-1β, IL-6, and IL-10 in rat serum. **C** ELISA was used to determine the concentrations of TNF-α, IL-1β, IL-6, and IL-10 in the supernatants of cell culture. Values are mean ± SEM and obtained from 3 independent experiments. ^*^P < 0.05, ^**^P < 0.01, ^***^P < 0.001

The activation of the vascular endothelial adhesion factor was accompanied by corresponding changes in markers of immune inflammation. Two weeks after the intervention, we performed qRT-PCR to evaluate the mRNA levels of inflammatory cytokines including TNF-α, IL-1β, IL-6, and IL-10 in the aortic tissues. The results revealed increases of TNF-α, IL-1β, and IL-6 levels after the exposure to AgNP compared to the control group (*P* < 0.01) (Fig. 4A). However, Se treatment effectively reversed the overexpression of these pro-inflammatory TNF-α, IL-1β, and IL-6 mRNA levels and induced an elevation in anti-inflammatory IL-10 (*P* < 0.001). Notably, similar trends were observed for inflammatory cytokine concentrations in rat peripheral blood plasma (Fig. 4B) and cell culture supernatants of HUVECs (Fig. 4C). These findings indicated that Se intervention was effective in reducing AgNP-induced inflammation of vascular endothelium both in *vitro* and *in vivo* conditions.

### Se Treatment Attenuated Oxidative Stress in AgNP-Administered Rats and HUVECs via the Nrf2/HO-1 Signal Pathway

**Fig. 5:**
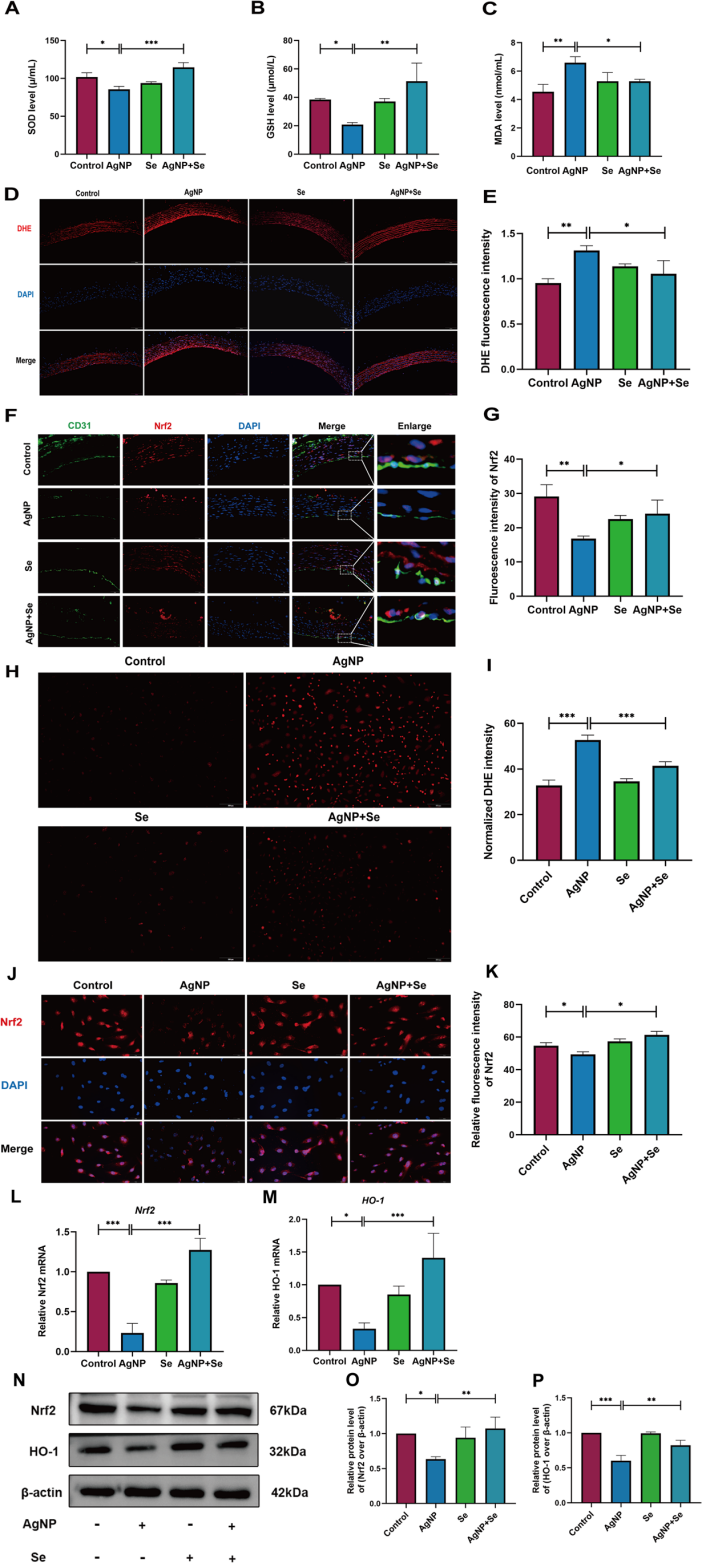
Se intervention prevented AgNP-induced oxidative stress by regulating Nrf2/HO-1 pathways in HUVECs. **A**–**C** Serum SOD, GSH activity, and MDA levels were respectively shown in the histograms. **D** Representative images of ROS levels in the thoracic aorta of rats in diverse groups (bars = 100 µm). **E** Quantification of ROS levels in rat thoracic aorta by fluorescence intensity. **F** Dual immunofluorescence staining for co-expression of Nrf2 and CD31 in aortic endothelial cells. Blue, nuclei; red, Nrf2; green, CD31; (bars = 50 µm). **G** Fluorescence intensity analysis of the Nrf2 in aortic tissues. **H** Representative images of intracellular ROS level (bars = 100 µm). **I** Measurement of intracellular ROS level through fluorescence intensity quantification. **J**–**K** Representative photomicrographs of immunofluorescence and quantitative analysis detection of Nrf2 (red) in HUVECs cells (bars = 50 µm). **L**, **M** The mRNA expression levels of Nrf2 and HO-1 in aortic tissues were analyzed by qRT-PCR. **N** Representative western blot images and the protein levels of Nrf2 and HO-1 in HUVECs (**O**, **P**). Values are mean ± SEM and obtained from 3 independent experiments. ^*^P < 0.05, ^**^P < 0.01, ^***^P < 0.001

In order to evaluate the underlying mechanisms of the Se protective effect, crucial oxidative stress indicators including levels of MDA, GSH, and SOD were firstly measured. The data obtained were depicted in Fig. 5A–C and demonstrated notable decreases in both SOD and GSH activities in the serum of the AgNP group compared to the control group (*P* < 0.05), along with a substantial increase in MDA level (*P* < 0.01). However, after Se treatment, abnormal SOD and GSH activities and MDA levels were corrected compared to the AgNP group (*P* < 0.05). Additionally, as illustrated in Fig. 5D and E**,** the fluorescence intensities of ROS in the AgNP intervention group appeared a dramatically increase compared to that in the control group (*P* < 0.01), whereas Se intervention exhibited a pronounced reduction in ROS fluorescence intensities as compared to the AgNP group (*P* < 0.05). The modulatory role of Se in AgNP-induced disorder of oxidative stress was further *in vitro* investigated in HUVECs. As shown in Fig. 5H and I, AgNP exposure resulted in excessive oxidative stress of HUVECs with a noticeable increase in ROS generation in comparison with the untreated cells (*P* < 0.001). Intriguingly, Se treatment obviously suppressed AgNP-induced ROS generation in HUVECs (*P* < 0.001). This section’s result further suggested that Se can attenuate the oxidative damage to vascular endothelial cells induced by AgNP exposure.

Furthermore, the Nrf2/HO-1 pathway has been considered to be crucial for cell survival by responding to oxidative stress. We subsequently evaluated the Nrf2 expression in rat aortic endothelial cells of diverse groups in rats (Fig. 5F, G) and HUVECs (Fig. 5J, K) by immunofluorescence staining. It was observed that Nrf2 expression was significantly decreased in the aortas of rats and HUVEC cells exposed to AgNP, but this was notably rectified after the treatment with Se. Besides, the results depicted in Fig. 5L and M demonstrated that the mRNA expressions of Nrf2 and HO-1 were significantly lower in the aortic tissues of the AgNP group than those in the control group (*P* < 0.05 for HO-1 and *P* < 0.001 for Nrf2). As expected, the expressions of the aforementioned mRNA molecules were markedly elevated in the AgNP+Se group in comparison with the AgNP group (*P* < 0.001). Further, western blot analysis was employed to identify the protein levels of Nrf2 and HO-1 in HUVECs (Fig. 5N–P). Noteworthy, Nrf2 and HO-1 levels were conspicuously reduced in the AgNP-exposed group compared to the control group, whereas all protein levels were elevated in the selenium-treated group. Collectively, these results indicated that selenium treatment upregulated the Nrf2 /HO-1 pathway in response to the oxidative stress induced by AgNP in both in *vivo* animal intervention experiment and *in vitro* cellular incubation experiment.

### Se Treatment Inhibited the Activation of the NLRP3 Inflammasome in AgNP-Administered Rats and HUVECs

**Fig. 6:**
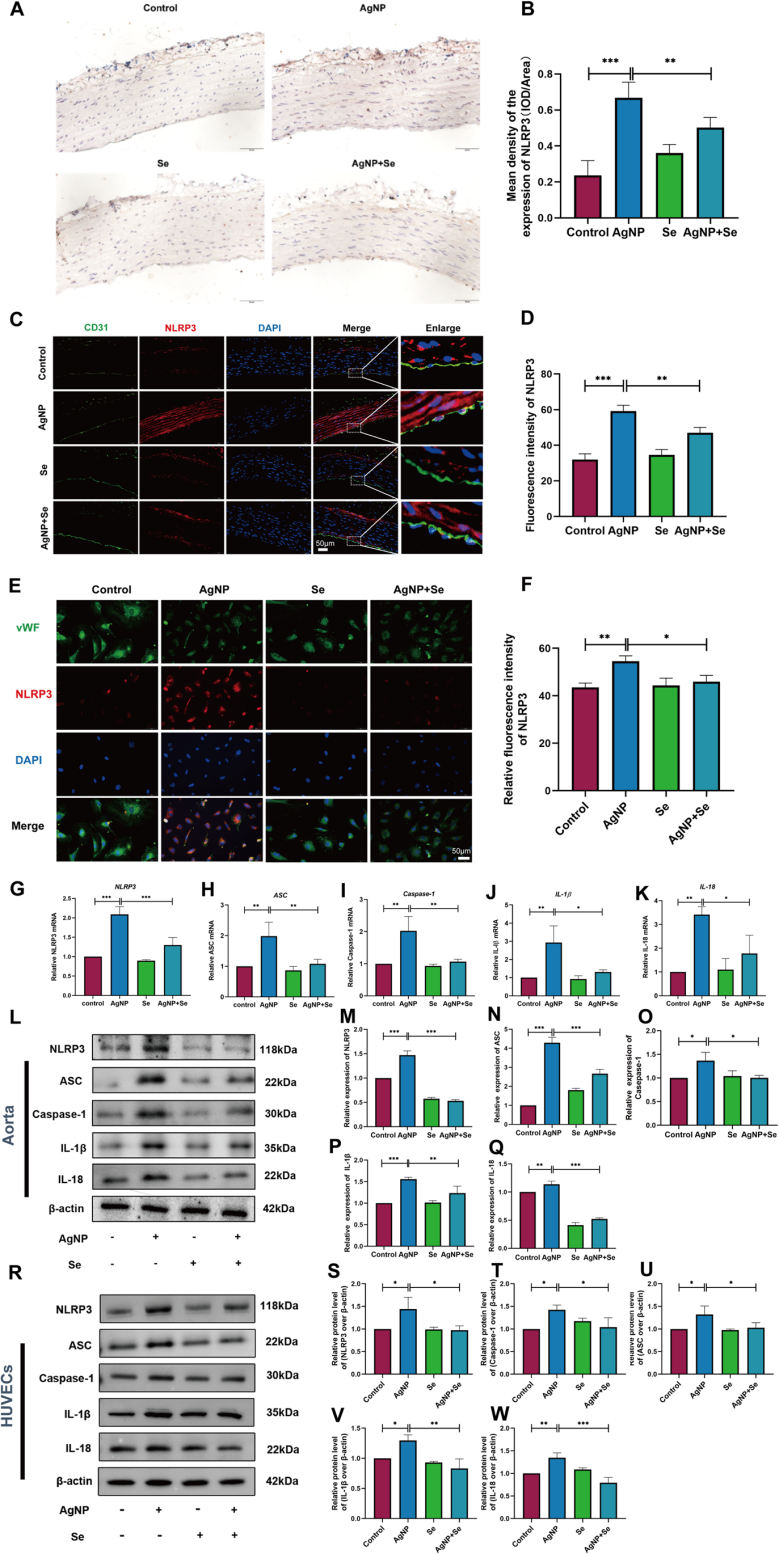
Se treatment inhibited the activation of the NLRP3 inflammasome in AgNP administered rats and HUVECs. **A**, **B** Representative photomicrographs of immunohistochemistry and quantitative analysis of NLRP3 in the aortic tissues, the positive cells were stained brown (bars = 50 µm). **C** Dual immunofluorescence staining for co-expression of NLRP3 and CD31 in aortic endothelial cells. Blue, nuclei; red, NLRP3; green, CD31 (bars = 50 µm). **D** Fluorescence intensity analysis of the NLRP3 in aortic tissue. **E** Dual immunofluorescence staining for co-expression of NLRP3 and von Willebrand factor (vWF) within the HUVEC cells. Blue, nuclei; red, NLRP3; green, vWF; (bars = 50 µm). **F** Fluorescence intensity analysis of the NLRP3 in HUVECs. **G**–**K** The mRNA expression levels of NLRP3 inflammasome, IL-1β, and IL-18 were represented. **L** Representative western blot images and the protein levels of NLRP3, ASC, Caspase-1, IL-1β, and IL-18 in the aortic tissues (**M**–**Q**) and the expressions (**R**) in HUVECs (**S**–**W**).Values are mean ± SEM and obtained from 3 independent experiments. ^*^P < 0.05, ^**^P < 0.01, ^***^P < 0.001

Our experimental results provided evidence of Se’s effectiveness on endothelial injury in the aortas of rats exposed with AgNP may partly attributed to the anti-inflammatory effect. Thus, to further reveal this pathogenesis, the critical role of NLRP3 inflammasome in the inflammatory reaction was verified by immunohistochemical analysis and showed more and thicker positive cellular staining during persistent AgNP exposure, which became dramatically weaker after Se intervention (Fig. 6A, B). Consistently, the fluorescence signal of NLRP3 interacting with CD31 in aortic endothelial cells was obviously aggravated in the AgNP group compared to the control animals (Fig. 6C–D). However, the fluorescence intensity at the junction of NLRP3 and CD31 was reduced after Se administration. In addition, immunohistochemical analysis of NLRP3 inflammasome in HUVECs showed similar results to those described above (Fig. 6E, F). The alterations observed in the AgNP group including the levels of NLRP3, ASC, Caspase-1, IL-18, and IL-1β mRNA expressions (Fig. 6G–K) and protein concentrations (Fig. 6L–W) were considerably higher in the AgNP-exposed group than in the control group. In contrast, Se treatment obviously suppressed AgNP-induced NLRP3 inflammasome (*P* < 0.05). The results of our study suggested that the occurrence of NLRP3 inflammasome assembly and subsequent associated endothelial cell damage were probably responsible for the consequence of exposure to AgNP. Therefore, the administration of Se was demonstrated to act as an inhibitory impact on the activation of NLRP3 inflammasome during sustained exposure of AgNP.

### Se Treatment Suppressed HMGB1/NF-κB Signaling Pathway in AgNP-Administered Rats and HUVECs

**Fig. 7:**
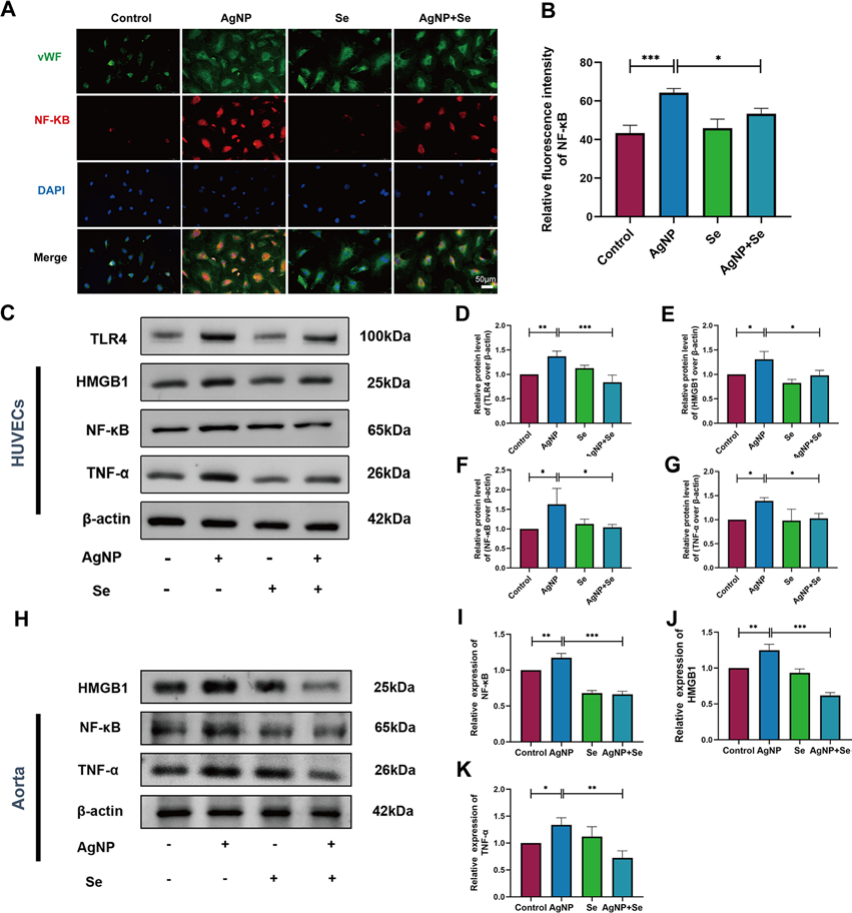
Se intervention suppressed HMGB1/NF-κB signaling pathway *in vitro* and *in vivo*. **A** Dual immunofluorescence staining for co-expression of NF-κB and vWF within the HUVEC cells. Blue, nuclei; red, NF-κB; green, vWF (bars = 50 µm). **B** Fluorescence intensity analysis of the NF-κB in HUVECs. **C** Representative western blot images and the protein levels of TLR4, HMGB1, NF-κB, and TNF-α in HUVECs (**D**–**G**). **H** Representative western blot images and the protein levels of HMGB1, NF-κB, and TNF-α in the aortic tissues (**I**–**K**). Values are mean ± SEM and obtained from 3 independent experiments. ^*^P < 0.05, ^**^P < 0.01, ^***^P < 0.001

**Fig. 8:**
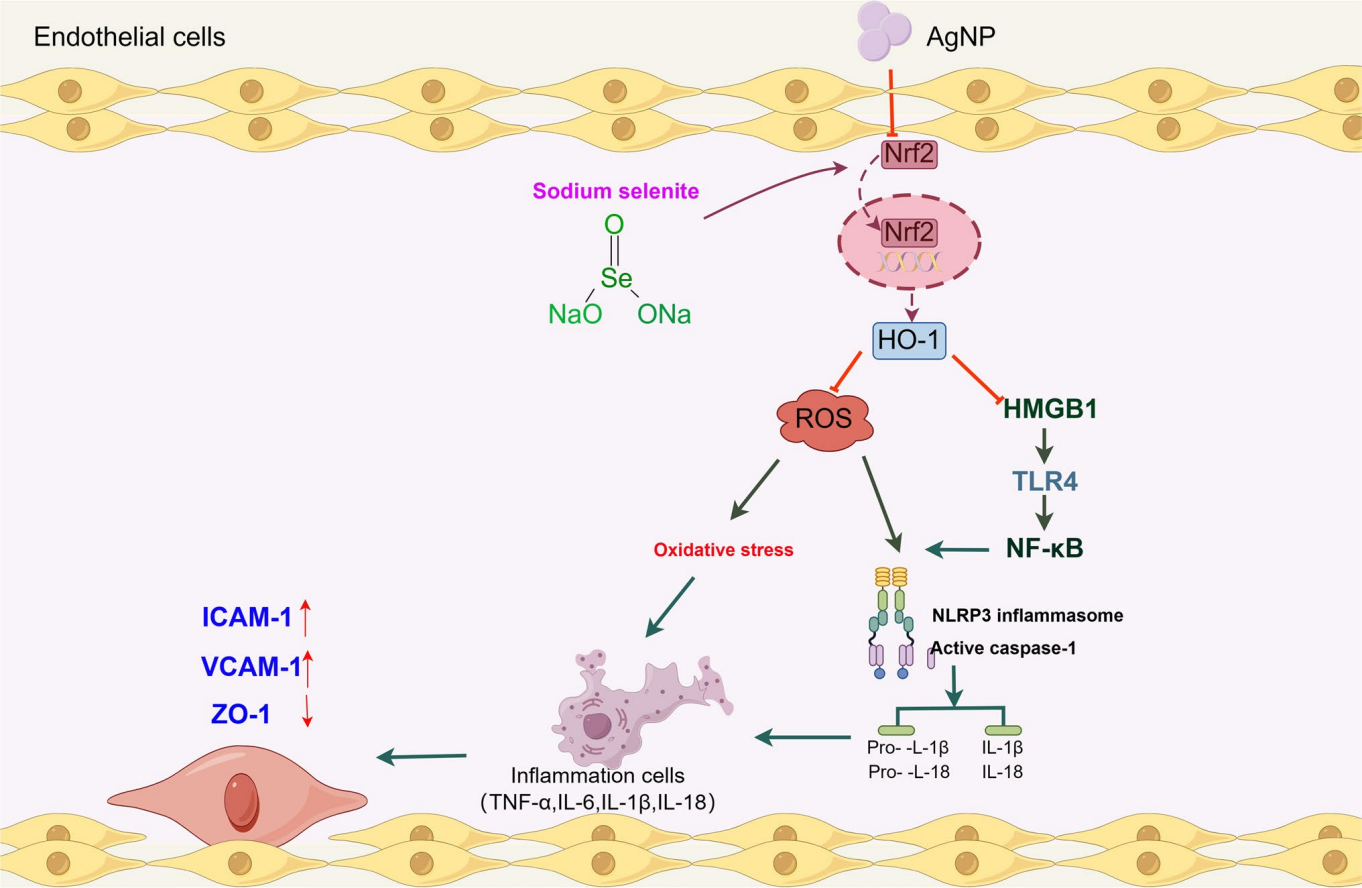
The protective effect of sodium selenite (Se) on silver nanoparticles (AgNP)-induced vascular endothelial cell injury and the underlying mechanisms. Se concomitantly alleviates HMGB1 and NF-κB secretion by activating the Nrf2/HO-1 signaling pathway. This activation helps to inhibit oxidative stress and ROS mediated NLRP3 inflammasome activation during AgNP-induced endothelial cell injury

HMGB1, known as high mobility group box1, represents a kind of damage-associated molecular patterns (DAMP) that stands for a late mediator in the case of severe inflammation affecting the blood vessels. Recent studies have provided evidence that HMGB1 has the ability to initiate the NF-κB signaling pathway during the occurrence of vascular inflammation response [19]. Thus, we speculated that the HMGB1/NF-κB pathway activation may potentially participate in the inhibitory effect of Se on AgNP-induced vascular endothelial cell inflammation. The NF-κB and von Willebrand factor (vWF) co-localization immunofluorescence staining of HUVECs are presented in Fig. 7A and B. The fluorescence intensity of NF-κB interacting with vWF in the AgNP group was significantly higher than that in the control group (*P* < 0.001), respectively. Whereas the fluorescence intensity at the interface of NF-κB and vWF was distinctly reduced in the AgNP+Se treated HUVECs compared to the AgNP group (*P* < 0.05). Western blot results verified that the protein levels of TLR4 (*P* < 0.01), HMGB1 (*P* < 0.05), NF-κB (*P* < 0.05), and TNF-α (*P* < 0.05) were respectively elevated in AgNP-exposed HUVEC cells (Fig. 7C–G) and rat aortic tissues (Fig. 7H–K) compared with the related controls. Nevertheless, the protein levels of HMGB1 (*P* < 0.001), NF-κB (*P* < 0.001), and TNF-α (*P* < 0.01) in the AgNP+Se group were remarkably downregulated in comparison with the AgNP group. Collectively, our observations demonstrated that the therapeutic intervention with Se treatment effectively suppressed the aortic inflammation induced by AgNP through inhibition of the HMGB1/NF-κB pathway (Fig. 8).

## Discussion

In the last few decades, AgNP exhibiting excellent antimicrobial activities has experienced rapid development in the food industry [1], textile products [20], medical fields such as medical imaging [21], new antibacterial drugs [22], drug delivery [2], and other fields. Although AgNP represent a wide application, it still appears toxic effects on respiratory and cardiovascular health [4, 23]. Therefore, appropriate countermeasures for AgNP-induced damage should be investigated. Our recent findings have shown that the myocardium and lung effects of AgNP-induced toxicity in rats were aggravated by the presence of AgNP in the lung [11, 12]. For this investigation, we implemented an identical dose of AgNP and experimental protocol. However, our focus was directed towards elucidating the mechanism of action in the aortic tissue. Our study devoted to investigating the protective effect of Se intervention against AgNP exposure in vascular endothelium, as well as the underlying mechanisms, through both in *vivo* and in *vitro* experiments.

We found that AgNP exposure induced inflammation and oxidative stress in vascular endothelium of rat thoracic aortas and caused damage to HUVEC cells. Moreover, we found that Se intervention could activate the Nrf2/HO-1 antioxidant stress pathway and subsequently ameliorate these toxic effects of AgNP.

As the most commonly medical nanoparticles, the general exposure route of AgNP includes the digestive tract, respiratory tract, skin, or even directly through blood vessels [24–27]. When AgNP enters the blood circulation, vascular endothelial cells are the first line to be directly affected, releasing inflammatory factors such as IL-8, intercellular adhesion molecule ICAM-1, and vascular cell adhesion molecule VCAM-1 for promoting adhesion and migration of monocytes and lymphocytes to the endothelium, as well as start the formation process of atherosclerotic plaque. Therefore, the overexpression of ICAM-1 and VCAM-1 is generally considered to be the key biochemical markers indicating vascular endothelial cell injury [28]. In our study, the exposure of AgNP dramatically increased the levels of ICAM-1 and VCAM-1, which was in agreement with the results of Shi et al. [29]. In addition, histological examination in the current research showed that AgNP induced morphological and structural damages of aortic tissue with the disordered smooth muscle cells and proliferated plenty of collagen fibers in the vascular wall. This result further confirms the damage caused by AgNP to the vasculature. Moreover, AgNP exposure increased the production of pro-inflammatory IL-1β, IL-6, and TNF-α, which in turn, further exacerbated endothelial inflammation and even dysfunction. In contrast, Se supplementation reversed vascular endothelial cell damage and aortic histopathology alterations, as well as alleviated inflammation, consistent with previous investigations [30, 31]. These suggested a protective effect of Se intervention against AgNP exposure-induced aortic endothelial injury.

Endothelial cells are crucial for maintaining vascular function and homeostasis, ensuring the structural integrity and selective permeability of the blood vessels. A significant indicator of heightened endothelial permeability in vascular dysfunction is the downregulation of ZO-1 and VE-cadherin [32]. An investigation conducted *in vivo* demonstrated that the intravenous administration of AgNP can be absorbed by the vascular endothelium, resulting in space between the normally tightly packed endothelial cells and discontinuous distribution of VE-cadherin. After the integrity of the vascular endothelium is damaged, endothelial cell leakage occurs, leading to inflammation in surrounding organs such as the liver, kidney, and lung [33]. AgNP exposure could also decrease the protein ZO-1 and significantly increase the blood-brain barrier permeability [34, 35]. Our research found that AgNP caused a significantly decrease in ZO-1 expression within the aortic tissue of rats and increased vascular permeability, the protective role of Se against vascular leakage was evidenced through the ZO-1 immunofluorescence staining and Evans blue test. These results indicated that endothelial cell dysfunction may result from the disturbance of tight junction proteins upon exposure to AgNP.

Endothelial barrier dysfunction has been associated with impaired endothelium-dependent vasodilatation [36]. It has been reported that in the isolated rat aortic ring vascular tension test, a low concentration of AgNP (45 nm) induces vasoconstriction, whereas a high concentration stimulates vasodilation [37]. eNOs produced by the vascular endothelium is a major endogenous vasodilator. AgNP can increase oxidative stress by reducing endothelial NO synthesis, resulting in decreased vascular reactivity [3] and impaired vascular contractile function. Consistent with these previous studies, our data demonstrated that AgNP stimulation caused significantly decreased levels of eNOs, as well as we also observed that the number of neovascular sprouts in the aortic ring of rats was apparently reduced after AgNP intervention compared to the control group. Notably, Se supplementation markedly improved eNOs activity and significantly increased angiogenesis, indicating that Se may promote NO synthesis by activating signaling pathways related to eNOs and play a crucial biological role in the process of angiogenesis [38, 39]. These findings supported the idea that exposure to AgNP could damage the function of vascular endothelial cells and contribute to the progression of vascular disease. Se treatment had the beneficial effects on AgNP-induced vascular endothelial dysfunction.

High levels of ROS have been identified as a key factor in vascular injury and dysfunction. Interestingly, an essential mechanism for AgNP has been considered to be oxidative stress [40, 41]. A recent study showed that AgNP significantly reduced mitochondrial transmembrane potential, resulting in the production of a high level of ROS [42]. We also observed that AgNP-induced thoracic aortic damage and reduced HUVEC viability were associated with activating oxidative stress. Notably, Se intervention reduced AgNP-induced ROS and MDA levels and improved the activity of SOD and GSH in this study. These suggested that Se could alleviate AgNP-induced vascular endothelial damage by inhibiting oxidative stress.

Several studies have demonstrated that the role of ROS was not only as a by-product of metabolism but also as an activator of the NLRP3 inflammasome, leading to disruption of endothelial function. The activation of the NLRP3 inflammasome leads to the recruitment and cleavage of procaspase-1, as well as the regulation of cytokines IL-1β and IL-18, ultimately causing inflammatory responses. In our present study, the results indicated that AgNP exposure induced NLRP3 inflammasome overactivation and increased NLRP3, caspase-1, and IL-1β protein levels. Intriguingly, Se supplementation could suppress the AgNP exposure-induced NLRP3 inflammasome and inhibited the increase of ROS in AgNP-exposed rats and HUVECs, this was consistent with findings from previous studies [43, 44].

HMGB1 is released from damaged cells, while the oxidative stress environment in damaged tissues promotes HMGB1 binding to Toll-like receptor 4 (TLR-4) to trigger a variety of inflammatory diseases. Recent studies have shown that HMGB1 can initiate an inflammatory response by upregulating the expression of ICAM-1 and VCAM-1 on endothelial cells, thereby, it promotes the adhesion and migration of white blood cells through endothelial cells to the sites of inflammation [45]. Moreover, the HMGB1 protein has been widely recognized for its ability to activate the NF-κB signaling pathway and NLRP3 inflammasomes [19, 46]. *In vitro* studies suggested that exposure to AgNP increased the activity of NF-κB in HUVECs [29], and TLR4 mainly contributed to the cytokine production induced by AgNP [47]. Therefore, HMGB1 is a key mediator in promoting inflammation. In this research, we observed the production of HMGB1, the stimulation of NF-κB, and the activation of NLRP3 inflammasome in diverse experimental groups. It is important to note that Se supplementation has the capability to suppressing HMGB1, NF-κB, and NLRP3 inflammasome levels in both aortic tissue and HUVECs induced by AgNP. This supports the notion that Se has the ability to alleviate inflammation-mediated endothelial cell damage by inhibiting the HMGB1/NF-κB/NLRP3 signaling pathway.

The Nrf2 signaling pathway is the major element in the resistance of cells to toxic injury and oxidative stress, and it has been demonstrated that Se can enhance its antioxidant properties through activation of the Nrf2 pathway [47, 48]. To explore the potential mechanisms underlying the Se against AgNP exposure-induced toxicity, we evaluated the potential protective role of the Nrf2/HO-1 signaling pathway in the efficacy of Se supplementation. Our study demonstrated that AgNP induced a decrease in the expression levels of Nrf2 and HO-1 in aortic vascular endothelium, respectively. Consistent with the findings of the current research, acute toxicity caused by AgNP was reported to decrease Nrf2 and HO-1 expression in lung tissues of ICR mice [49]. Therefore, AgNP-induced ROS generation is a direct consequence of the decreased Nrf2 and HO-1 expression. HO-1 is an important anti-inflammatory and antioxidant enzyme that is essential for blocking HMBG1 secretion and to nuclear translocation, related evidence suggests that HMGB1 release increases with HO-1 deletion [50]. Our data suggest that Se treatment effectively reversed AgNP-induced reductions in Nrf2 activity and HO-1 content, it also concomitantly reduced MDA and ROS levels, inhibited NF-κB secretion and NLRP3 inflammasome activation, thereby rendering thoracic aorta and HUVECs resistant to AgNP-induced oxidative and inflammatory damage. The anti-inflammatory activity of Se treatment may be attributed to the inhibition of HMGB1 activation and upregulation of Nrf2/HO-1 expression.

## Conclusion

Our study provides further proof that Se supplementation can effectively reduce HMGB1and NF-κB secretion. This reduction is achieved through activation of the Nrf2/HO-1 signaling pathway, which in turn inhibits oxidative stress and ROS-dependent NLRP3 inflammasome activation in AgNP exposure-induced vascular endothelial toxicity.

### Supplementary Information

Below is the link to the electronic supplementary material.

Supplementary file1 (DOCX 583 KB)

## Data Availability

All data generated or analyzed during this study are included in this published article.
