# Supplementary material for: Sodium Selenite Ameliorates Silver Nanoparticles Induced Vascular Endothelial Cytotoxic Injury by Antioxidative Properties and Suppressing Inflammation Through Activating the Nrf2 Signaling Pathway
Source: Biol Trace Elem Res. 2023 Dec 27;202(10):4567–85. doi: 10.1007/s12011-023-04014-2 (PMC11339151; doi:10.1007/s12011-023-04014-2)

**Supplementary material**

**Sodium selenite ameliorates silver nanoparticles induced** **vascular endothelial cytotoxic injury by antioxidative properties and suppressing inflammation through activating the Nrf2 signaling pathway**

**Supplementary material as follows**

**Table S1**

The dietary nutrients in this study were formulated according to GB 14924.3

| Lysine (g/kg) | 13.5 |
| --- | --- |
| Methionine + Cystine (g/kg) | 7.6 |
| Arginine (g/kg) | 11.9 |
| Histidine (g/kg) | 6.0 |
| Tryptophan (g/kg) | 3.6 |
| Phenylalanine + Tyrosine (g/kg) | 14.9 |
| Threonine (g/kg) | 8.7 |
| Leucine (g/kg) | 14.5 |
| Isoleucine (g/kg) | 9.5 |
| Valine (g/kg) | 10.5 |
| Vitamin A (IU/kg) | 13692 |
| Vitamin D (IU/kg) | 1339 |
| Vitamin E (IU/kg) | 115.4 |
| Vitamin K (mg/kg) | 5.5 |
| Vitamin B1 (mg/kg) | 15 |
| Vitamin B2 (mg/kg) | 17.4 |
| Vitamin B6 (mg/kg) | 10 |
| Vitamin B12 (mg/kg) | 0.035 |
| Nicotinic acid (mg/kg) | 74.9 |
| Pantothenic acid (mg/kg) | 32.7 |
| Folic acid (mg/kg) | 7.50 |
| Biotin (mg/kg) | 0.174 |
| Choline (mg/kg) | 1360 |
| Magnesium (g/kg) | 2.27 |
| Potassium (g/kg) | 6.53 |
| Sodium (g/kg) | 3.11 |
| Iron (mg/kg) | 195 |
| Manganese (mg/kg) | 105 |
| Copper (mg/kg) | 14.1 |
| Zinc (mg/kg) | 55 |
| Iodine (mg/kg) | 0.55 |


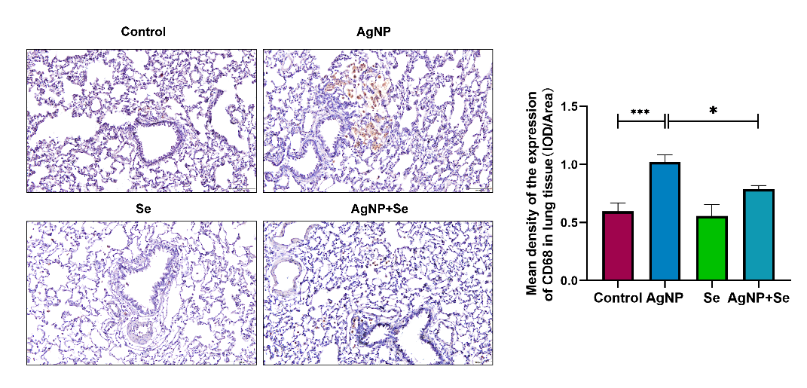
**Fig S1.** Representative photomicrographs of immunohistochemistry and quantitative analysis showing the detection of CD68 in the lung tissue, the positive areas were stained in brown (Bars =100 μm)**.** Values are mean ± SEM and obtained from 3 independent experiments. **P* < 005, ***P* < 0.01, ****P* < 0.001.

**Fig S2+S3.** Cell viability of HUVECs were cultured with AgNP or Se at different concentrations for 24 h tested by CCK-8 assay (n = 3 per group). Values are mean ± SEM and obtained from 3 independent experiments. **P* < 005, ***P* < 0.01, ****P* < 0.001.


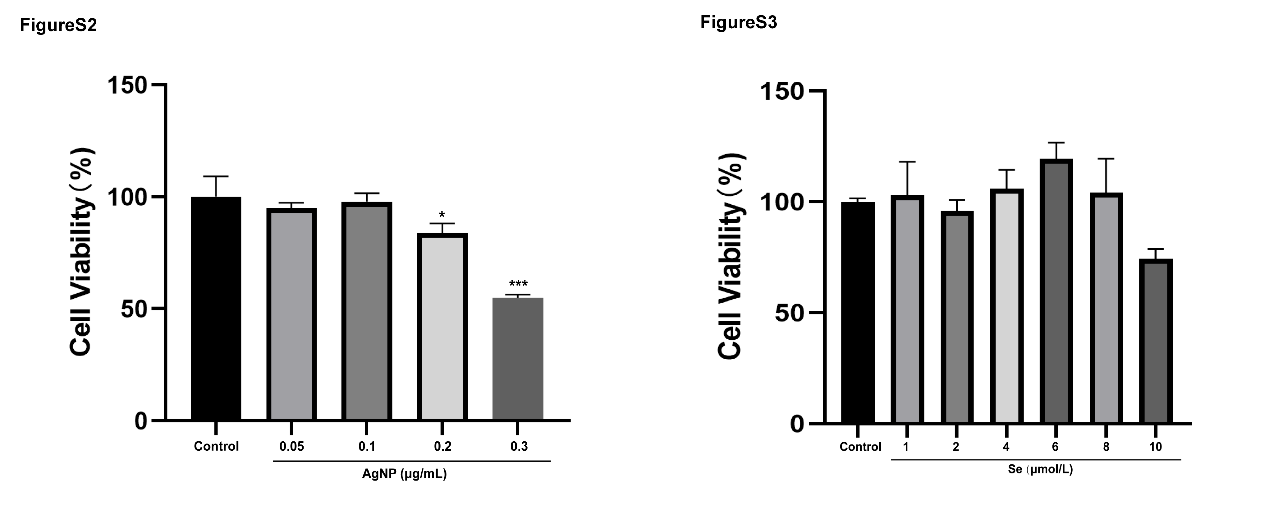


**Fig S4.** Quantitation of Evans blue extravasation in lung tissue. Values are mean ± SEM and obtained from 3 independent experiments. **P* < 005, ***P* < 0.01, ****P* < 0.001.


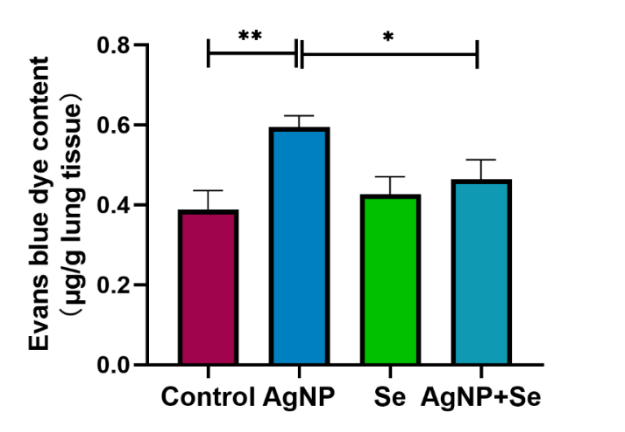

Supplement: Supplementary file 1 — Supplementary file1 (DOCX 583 KB) [file 12011_2023_4014_MOESM1_ESM.docx]
